# Exploring the use of social network analysis methods in process improvement within healthcare organizations: a scoping review

**DOI:** 10.1186/s12913-024-11475-1

**Published:** 2024-09-05

**Authors:** Troy Francis, Morgan Davidson, Laura Senese, Lianne Jeffs, Reza Yousefi-Nooraie, Mathieu Ouimet, Valeria Rac, Patricia Trbovich

**Affiliations:** 1 Institute of Health Policy, Management and Evaluation, Dalla Lana School of Public Health, University of Toronto, Toronto, Canada; 2 HumanEra, Research and Innovation, North York General Hospital, Toronto, ON Canada; 3 Program for Health System and Technology Evaluation, Toronto General Hospital Research Institute, University Health Network, Toronto, ON Canada; 4 Department of Public Health Sciences, University of Rochester, New York, USA; 5 Department of Political Science, Université Laval, Quebec, Canada

**Keywords:** Social network analysis, Process improvement, Quality improvement, Healthcare organizations, Patient safety, Organizational structure, Team performance

## Abstract

**Background:**

Communication breakdowns among healthcare providers have been identified as a significant cause of preventable adverse events, including harm to patients. A large proportion of studies investigating communication in healthcare organizations lack the necessary understanding of social networks to make meaningful improvements. Process Improvement in healthcare (systematic approach of identifying, analyzing, and enhancing workflows) is needed to improve quality and patient safety. This review aimed to characterize the use of SNA methods in Process Improvement within healthcare organizations.

**Methods:**

Relevant studies were identified through a systematic search of seven databases from inception - October 2022. No limits were placed on study design or language. The reviewers independently charted data from eligible full-text studies using a standardized data abstraction form and resolved discrepancies by consensus. The abstracted information was synthesized quantitatively and narratively.

**Results:**

Upon full-text review, 38 unique articles were included. Most studies were published between 2015 and 2021 (26, 68%). Studies focused primarily on physicians and nursing staff. The majority of identified studies were descriptive and cross-sectional, with 5 studies using longitudinal experimental study designs. SNA studies in healthcare focusing on process improvement spanned three themes: Organizational structure (e.g., hierarchical structures, professional boundaries, geographical dispersion, technology limitations that impact communication and collaboration), team performance (e.g., communication patterns and information flow among providers., and influential actors (e.g., key individuals or roles within healthcare teams who serve as central connectors or influencers in communication and decision-making processes).

**Conclusions:**

SNA methods can characterize Process Improvement through mapping, quantifying, and visualizing social relations, revealing inefficiencies, which can then be targeted to develop interventions to enhance communication, foster collaboration, and improve patient safety.

**Supplementary Information:**

The online version contains supplementary material available at 10.1186/s12913-024-11475-1.

## Introduction

Adverse events, including medical errors, diagnostic errors, and preventable complications, continue to affect millions of patients globally, leading to severe morbidity, mortality, and substantial avoidable healthcare costs [1, 2]. Among the many factors contributing to avoidable adverse events, breakdowns in communication have been identified as a leading cause [3–5]. Lapses in communication during care coordination and patient handoffs can lead to inadequate patient follow-up, delayed care, increased healthcare costs, and provider burnout, leading to an increased risk of adverse events [4, 6].

Many studies have highlighted that investigating the underlying causes and consequences of poor communication is necessary to improve the delivery of high-quality care [3, 4, 6, 7]. However, a large proportion of studies investigating communication in healthcare organizations lack the necessary understanding of social structures (interconnected relationships of social groups e.g., who speaks to who, for what purpose, using what mechanism) and coordination structures (e.g., how information gets transferred or transitioned between people or services) to make meaningful improvements and reduce adverse events [8, 9]. For example, the surgical safety checklist (SSC) is a tool meant to enhance patient safety by coordinating care delivery and improving inter-professional communication [10]. Yet, many studies report conflicting results on the impact of the SSC due to a lack of mutual understanding of communication among team members (e.g., who is responsible for leading a specific checklist pause point) and coordination (e.g., what team members should be present during specific pause points) structures (11–13). Effective communication among healthcare providers is challenging due to the complex nature of tasks performed and the numerous healthcare providers embedded within hierarchical structures. While the effective use of Process Improvement or Quality Improvement (QI; framework to systematically improve processes and systems in healthcare) interventions rely on understanding the social interactions and relationships within organizations, little attention has been paid to how social networks can be used to improve the effectiveness of communication and coordination in healthcare.

A social network is a set of social entities, actors or nodes (individuals, groups, organizations) connected by similarities, social relations, interactions, or flows (information) [14]. Analyzing professional communication structures (e.g., observed formal advice-seeking or giving related to work situations) within healthcare organizations’ social networks is important in understanding how best to inform interventions by identifying which network structures promote or inhibit behavior change [15]. The use of social network analysis (SNA) can provide insight into the social relationships, interactions, and tasks involved within sociotechnical systems. SNA metrics are quantitative measures used to analyze the structure, relationships, and dynamics within social networks through quantifying network behavior [16]. Network metrics reflect *centrality*, which refers to a family of measures where each represent different conceptualizations of nodal importance within a network, and *cohesion* measures, which examine the extent to which nodes within a network are connected [14, 17]. These metrics provide an understanding of the structure of social networks through identifying influential nodes, information flow, communities, and cliques [18]. SNA has been shown to improve professional communication and interprofessional relationships by revealing gaps in communication and identifying influential social entities and communication channels [14, 15, 19]. By indicating which social entities are effective in the flow of communication, organizations can leverage their skills to disseminate important information effectively and foster positive inter-professional relationships [19, 20]. Additionally, through identifying gaps in communication between different teams or departments organizations can work to prevent misunderstandings, adverse events, and the duplication of efforts resulting in a more collaborative work environment with stronger interprofessional relationships [14, 21]. Through understanding social networks, SNA can be effective in designing, implementing, and evaluating interventions needed to improve professional communication and coordination in healthcare [15, 22].

The aim of this review was to characterize the existing literature to assess SNA methods ability to identify, analyze, and improve processes (Process Improvement) related to patient care within healthcare organizations.

## Methods

The scoping review was conducted using Arksey and O’Malley’s modified six-step framework [23, 24]. The Preferred Reporting Items for Systematic Reviews and Meta-Analyses extension for Scoping Reviews (PRISMA-ScR) standards were used to guide the reporting of this review [25]. The PRISMA-ScR checklist is shown in the Appendix.

### Information sources and search strategy

In collaboration with a research librarian (JB), relevant studies were identified through a systematic search of the MEDLINE (Ovid), Embase, Psychinfo, AMED (Allied and Complementary Medicine), CINAHL, Cochrane Library and Web of Science databases from inception – 16 October 2022. The database search was supplemented with hand searching of reference lists of included reviews. Grey literature was searched using Google Custom Search Engine strategies to narrow search results and allow for more targeted results [26, 27]. Searched websites included the International Network for Social Network Analysis, American Evaluation Association Social Network Analysis Technical Interest Group, and the International Sunbelt Social Networks Conference proceedings archives. The search strategy for the social network analysis concept was adapted from Sabot et al.’s systematic review of Social Network Analysis and healthcare settings [22]. Truncation search terms were used to search inclusive and key terms for these concepts can be found in the supplemental appendix.

### Eligibility criteria

A screening checklist developed by Sabot et al., 2017 was modified to guide the review of this study [22, 28]. A “no” response to any of the study inclusion criteria (Appendix) was a reason for exclusion from the scoping review. “Healthcare providers” were classified as physicians, physician’s assistants, nurses, midwives, pharmacists, pharmacy technicians, clinical officers, counselors, allied health professionals, and other individuals involved in professional networks (e.g., administrative support staff, management). “Professional communication” was defined as observed formal professional advice-seeking or giving related to hypothetical or actual work situations or patients [22]. Healthcare organizations were defined as a building or mobile enclosure in which human medical, dental, psychiatric, nursing, obstetrical, or surgical care is provided. Healthcare organizations can include but are not limited to, hospitals, nursing homes, limited care facilities, medical and dental offices, and ambulatory care centers [29]. Studies had to report the use of SNA in the design of the study (e.g., social network mapping, evaluation of network properties or structure, or analysis of network actors) [22]. Additionally, to be included studies were required to use systematic data-guided activities (e.g., aims and measures) to achieve improvement or use an iterative development and testing process (i.e., Lean Management, Six Sigma, Plan-Do-Study-Act (PDSA) cycles, or Root Cause Analysis) [30, 31]. Studies where network relations were defined solely by patient sharing were excluded, as this only predicts person-to-person communication in a minority of instances [32]. Abstracts and conference proceedings were considered if details of their methodology and results were published. No limits were placed on study design, language, or publication period.

### Study selection and screening process

Study selection and screening employed an iterative process involving searching the literature, refining the search strategy, and reviewing articles for study inclusion. The titles and abstracts of all identified references were independently examined for inclusion by three reviewers (T.F, M.D, and L.S) using the Covidence software platform for systematic reviews [33]. Full texts of potentially eligible studies were retrieved by the reviewers (T.F, M.D, and L.S), who determined study eligibility using a standardized inclusion screening checklist. Inter-rater reliability was assessed at each phase of the scoping review between reviewers and disagreements were resolved by consensus with input from a fourth author (L.J).

### Charting the data

Data from eligible full-text studies was charted by the reviewers (T.F, M.D, L.S) independently using a standardized data abstraction form in Covidence to obtain key items of information from the primary research reports. Discrepancies among reviewers were resolved by consensus. The data abstraction form captured information on key study characteristics (e.g., author, year of publication, location of study, study design, aim of study, type of healthcare facility/provider), SNA-related information (e.g., SNA purpose, data collection methodology, software, SNA metrics) and reported on the implications of using SNA (e.g., social network mapping, assessment of network members or structures).

### Collating, summarizing, and reporting the results

A narrative synthesis was performed to describe the study characteristics, SNA methodology, and SNA metrics. The stages of the narrative synthesis included: (1) developing the preliminary synthesis, (2) comparing themes within and between studies, and (3) thematic classification [34]. Detailed text data on SNA characteristics and implications were reviewed, re-categorized, and analyzed thematically. In line with our objectives, the thematic analysis focused on identifying SNA methods used to improve communication and coordination in healthcare organizations. To categorize the approaches, we conducted further distillation of overarching approaches. We took notes throughout the review and analysis stages, documenting emerging trends and ideas to facilitate further review and discussion among the review team. The extracted data was tabulated in descriptive formats and narrative summaries were provided.

## Results

**Fig. 1:**
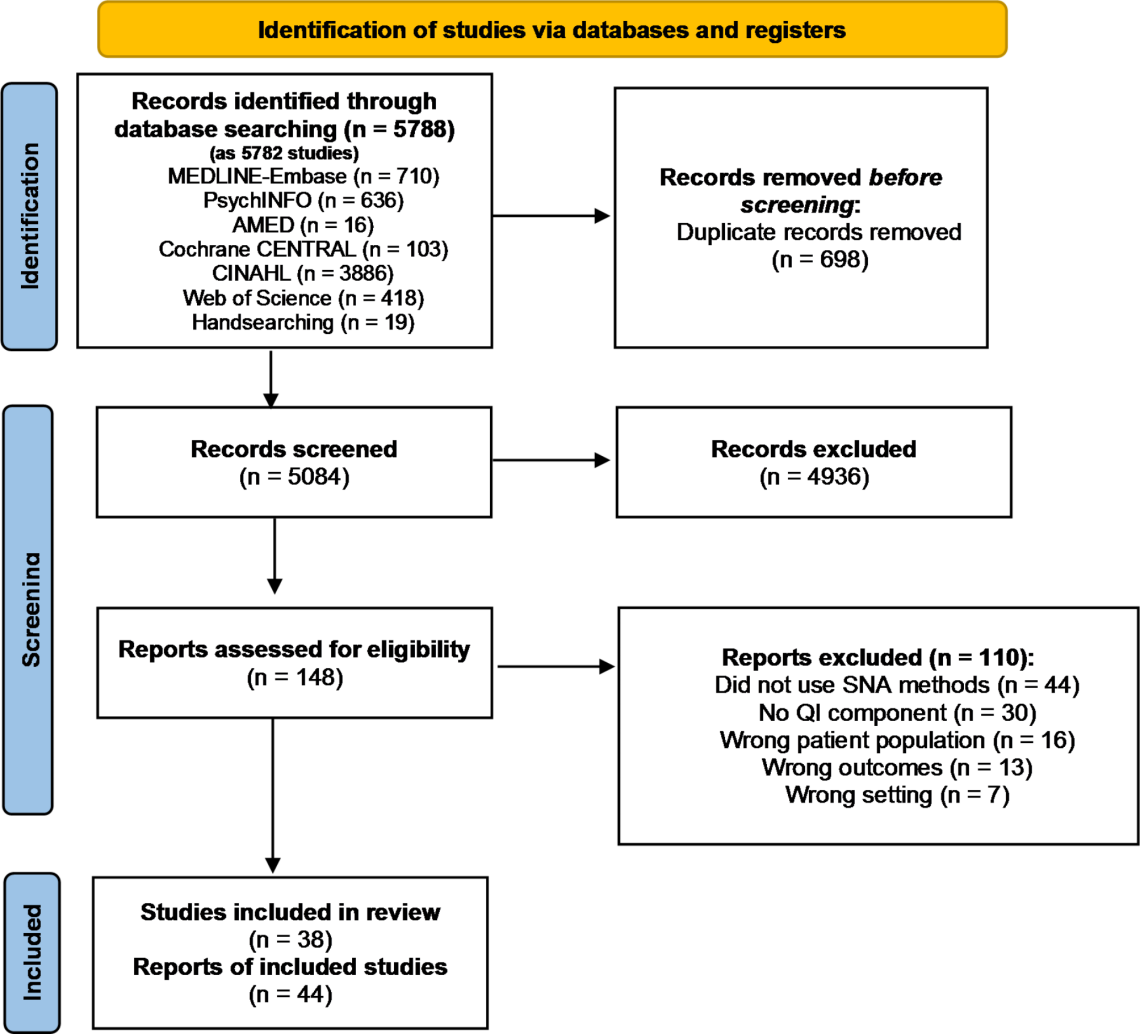
PRISMA-ScR flow diagram

The literature search generated 5084 potentially eligible studies after deduplication, of which 4936 were excluded based on title and abstract, leaving 148 full-text articles to be reviewed. The PRISMA-ScR flow diagram outlining the breakdown of studies can be found in Fig. 1. Upon full-text review, 44 reports of 38 studies were included for data abstraction. Six studies [4, 35–39] had multiple records and were truncated into single studies.

## Study characteristics

The characteristics of the included studies are shown in Table 1. Many studies were recently published between 2015 and 2021 (26, 68%) and were primarily located in the United States (26, 68%). 67% of studies occurred within a hospital (25, 66%) and most studies (15, 39%) were set in Internal medicine (gastroenterology, oncology, cardiology, nephrology, respirology, telemetry, or acute care). Studies employed multidisciplinary healthcare providers, however many studies focused on physicians (endocrinologists, oncologists, plastic surgeons, neurologists, anesthesiologists, intensivists, generalists; 27, 71%) and nursing staff (registered nurse, nurse practitioner, practical nurse; nursing assistants; 27, 71%). Most studies employed an observational study design, with 5 studies utilizing longitudinal quasi-experimental design [40–44]. Five studies used mixed-methods designs [35, 36, 45–47] with integrated qualitative and quantitative data, and a further 6 studies used multi-method designs [48–53] using a combination of independent qualitative and quantitative data. Twenty-four studies reported using quantitative data only [3, 4, 6, 40–43, 54–70] and the remaining 2 studies used qualitative methods [71, 72].

**Table 1:** Characteristics of included studies

Characteristics	Total *n* (%)
	(*n* = 38)
***Publication Date***	
2005–2009	4 (11)
2010–2014	8 (21)
2015–2021	26 (68)
***Study Location***	
United States	26 (68)
Australia	5 (13)
Israel	2 (5)
Other (Canada, UK, France, Italy)	5 (13)
***Type of Healthcare facility***	
Hospital	25 (66)
Medical Clinic	12 (31)
Nursing home	1 (3)
Healthcare setting*	
Internal medicine	15 (39)
Primary care practices	11 (29)
Surgical unit	5 (13)
Critical care unit	4 (11)
Other (ED, PACU, Public health, Psychiatry)	6 (16)
Type of healthcare provider*	
Physicians	27 (71)
Nursing staff	27 (71)
Corporate staff	11 (29)
Pharmacist	5 (13)
Lab technicians	3 (8)
Physician Assistant	2 (5)
Other (Dietitians, Social workers, Psychologists, OT)	10 (26)
***Study Design***	
Mixed-method (integration)	5 (13)
Multi-method (independent)	6 (16)
Quantitative only	25 (66)
Qualitative only	2 (5)

Table 2 provides an overview of the aims and findings of the included studies and Table 3 outlines the use of SNA methodology and reflects the data collection methods, software, and SNA metrics included in each study. A wide range of network visualization software was used with studies giving preferences towards UCINET [36, 40, 48, 54, 57–59, 66–68, 70, 72, 73], Organization Risk Analyzer (ORA) [4, 55, 74, 75], and Open-Sourced R Software [42, 49, 53, 63, 65, 76]. Five out of the 38 studies did not visualize their networks through social network mapping and only provided a descriptive assessment of network structures or analysis of network members [3, 40, 57, 68, 76]. Two studies did not explicitly report SNA metrics [47, 61]. Table 4 provides a comprehensive breakdown of the SNA metrics selected in each study and their application to healthcare networks. There were many network metrics used throughout the studies, however, most studies primarily employed Degree Centrality, Betweenness Centrality, and Density. Twenty-six studies used Degree Centrality as a measure of reach and importance [3, 4, 6, 35, 36, 41, 43–46, 48, 49, 51, 54–59, 62–65, 67, 69, 70], 20 studies used Density to measure network cohesion [6, 35, 36, 41, 43–45, 48, 53–55, 57, 58, 62, 63, 69–72, 77], and 19 studies used Betweenness Centrality as a measure of influence and brokerage [3, 4, 36, 44–46, 49, 51, 52, 55–57, 59, 60, 62, 63, 65, 66, 69].

**Table 2:** Summary of included studies

Author, date	Study aims	Findings
Hossain 2012	To model coordination within emergency departments through social network measures, defining measures of social networks and coordination to learn the relationship between them	Social networks and coordination are related within the emergency department. As emergency department density increases, patient triage wait times decreased. As degree increase, patient wait times increase. Centrality and the quality of coordination in the emergency department are linked. As team communication increases, patient revisits decreased
Effken 2011	To identify patient care unit communication patterns associated with patient safety and quality outcomes	Utilized ORA software for healthcare research and the relationship of nursing unit communication patterns to patient safety and outcomes. Except for falls and adverse drug events, more communication was associated with better patient outcomes
Benham-Hutchins 2010	To learn more about how healthcare providers communicate and exchange patient clinical information during patient handoffs (transfers) between units in an acute care setting	The network patterns that emerged uncovered the overlapping use of communication methods. No professional role dominated or information flow; instead each handoff network exhibited emergent non-linear communication patterns
Salwei 2019	To understand how the Venous thromboembolism (VTE) prophylaxis team adapts as the complexity in the process changes; we do this by using social network analysis (SNA) measures	A mutually exclusive relationship between an increase in the number of people on the team and an increase in reciprocity and density. VTE prophylaxis care teams adapted by increasing the roles, activities, and interactions among the team or by increasing two-way communication and discussion between team members
Nengliang 2018	To use the access-log data from the EHR to construct the network of the healthcare professionals providing cancer care to one subset of patients at an academic medical center	This study demonstrated that the access-log data from EHR could be used to describe the network structure of care delivered to patients with cancer. Social network analysis can identify structural characteristics of networks that emerged from interactions between providers mediated by their access to patients’ EHRs
Uddin 2012	To understand how the different network structures of PCN affect hospitalization cost and readmission rate? and what structural properties of PCN are related to hospitalization cost and readmission rate? And how they affect hospitalization cost and readmission rate?	Degree centrality and network density of physician collaboration network are negatively correlated with hospitalization cost and readmission rate. Conversely, betweenness centrality positively correlates with hospitalization cost and readmission rate. Distance was found to positively correlate with hospitalization costs but negatively correlates with readmission rate
Parnell 2020	To (1) determine the association between nurse managers leadership practices and quantifiable social network properties of employees they lead, (2) determine the association between social network properties within inpatient nursing units and outcomes reflected in their National Database of Nursing Quality Indicators (NDNQI) data, and (3) examine the combined relationships of leadership practices and social network properties with unit-level NDNQI data	Aim 1: Indicated that social network connectedness is associated with leaders who openly recognize contributions and show appreciation. Aim 2: No correlation between social network properties and outcomes. Aim 3: cohesive social networks under supervision of managers who exhibit a high frequency of leadership practices may reduce patient falls on their units
Grippa 2018	To explore possible factors impacting team performance in healthcare, by focusing on information exchange within and across hospital boundaries	Results indicate that highly effective teams were more inwardly focused and less connected to external members. Furthermore, highly recognized teams communicated frequently but, less intensely than the others
Moore 2014	To gain insight into the social and relational dynamics of change in the context of health system reform and how they act to impact performance	SNA techniques provided a complementary view of role dynamics within a network that can be used to inform a more strategic approach to change alongside relational coordination
Sarti 2020	To evaluate the implementation of TGLNs (TGLN) Physician Leadership Model by examining critical implementation process variables (education/training, communication, satisfaction, participation and reach)	Social network analysis metrics, particularly participation and reach, indicated the Physician Leadership Model was dense at baseline. Hospital Donation Physicians (HDP) reported communication to be facilitated by their connections to their Regional Medical Leads. HDPs reported that intended outcomes were met
Rangachari 2019	To describe the structure of inter-professional knowledge exchange (or the patterns of connections among Social Knowledge Networking participants) related to EHR MedRec, during the 1-year SKN period (i.e., who spoke to whom), which in turn, provided a foundation for collective learning and practice change (i.e., MU of EHR MedRec) at AU Health	Results revealed that three of the five SKN moderators played a strong “collective brokerage” role in facilitating inter-professional knowledge exchange related to EHR MedRec. They played complementary roles in reinforcing best-practice assertions, providing IT system education, and synthesizing collective learning moments
Loveless 2015	To apply an innovative multimodal analytic approach that combines formal epidemiologic analysis, process evaluation and social network modeling of stroke care teams to describe and improve the critical system characteristics of high performing units in real world hospital systems	Critical path analysis and network mapping of the individual stroke team network interactions, demonstrated associations between the team structures and process/clinical outcomes
Bevc 2012	To assess how the hospital-based public health epidemiologists (PHE) program in North Carolina facilitates the exchange of public health surveillance information	Results identify a tendency for PHEs to serve as an intermediary between Local Hospital Departments and hospitals, with a high measure of degree centrality by LHDs and a low frequency of brokerage among hospitals
Samarth 2009	To observe communication patterns in social networks at the clinical workplace in the Post-Anesthesia Care Unit (PACU)	This analysis demonstrated a linkage between social network patterns in the PACU and workflow process efficiency. Manual workflow processes at PACU necessitate a hierarchical communication (star) structure that suffers from inherent bottlenecks causing buildup of OUT waitlist thereby resulting in patient delays
Boyer 2010	To describe relationships among healthcare professionals in a French public hospital using social network analysis (SNA) and to improve health service quality by strengthening health service management and leadership	Physicians had the highest scores for the three indicators. Older age was associated with higher centrality and clique number scores. Transversal activity was associated with higher scores than other specific activities (hospitalization, ambulatory care), except for emergency care
Rangachari 2008	To identify strategies not only for improving hospital coding performance but also for the hospital organization to adapt to the changing environment of quality reporting	This study finds that good-coding performance is systematically associated with a knowledge sharing network structure rich in brokerage and hierarchy rather than in density. This study suggests that to improve hospital coding performance, senior administrators must undertake proactive and unceasing efforts to coordinate knowledge exchange across physician and coding subgroups and connect these subgroups with the external environment
Westbrook 2007	To conceptualize an evaluation model reflecting the need for health care organizations to understand (and thus have the capacity to work on) the impact of the introduction of computerized physician order entry (CPOE) systems on both the technical and the social systems within organizations	Results show that on average there is a low frequency of interaction between staff members in terms of seeking advice regarding medication decisions, tasks and asking for help to solve work-related problems, and in socializing. Interactions that occur are mainly between members of the same professional group: nurses - nurses and doctors - doctors
Meltzer 2010	To develop a systematic approach to quantitatively describing the social environment within healthcare organizations, and to develop general principles based on SNA metrics for constructing quality improvement teams that will effectively disseminate interventions and effect behavior change	It is hypothesized that external connections may be most important when the collection or dissemination of information is the biggest priority, while the relationship of team members to each other may be most important when internal coordination, knowledge sharing, and within-group communication are desired
Spitzer-Shohat 2019	To investigate how the social network and structural ties among primary-care-clinic team members relate to their perceived team effectiveness (TE), in a large-scale disparity reduction intervention in Israel’s largest insurer and provider of services	Clinics with strong intra-clinic density and high clinic–subregional-management density were positively correlated with perceived TE. Additionally, clinic in-degree centrality was also positively associated with perceived TE
Hurtado 2020	To examine whether the champions pilot program was associated with 12-month improvements in self- reported indicators such as (1) use of equipment, (2) safety performance, (3) safety climate, and (4) safety sup- port. Afterward, we tested whether these improvements were greater among workers who indicated champions as a source of Safe patient handling and mobility (SPHM) advice compared with workers who did not	Results showed significant improvements in equipment use, safety participation, and safety compliance among workers who would seek SPHM advice from champions
Steitz 2019	To advance the understanding of provider coordination by evaluating the scope of electronic communication between providers treating breast cancer patients at a single institution	This study found that approximately 10% of relationships through shared patient were also involved in secure messaging. Medical oncologists had the largest overlap between networks across all specialties, suggesting that medical oncologists are key to coordinating care across all providers associated with a patient
Pepin 2019	To evaluate the impact of a leadership position for knowledge translation in occupational therapy in the first 2.5 years	The Lead Research Occupational Therapist moved from the periphery to the centre of the evidence-based practice social network. Improved awareness of other clinicians deploying evidence-based practice was observed, and the frequency of interaction between clinicians increased
Altalib 2019	To demonstrate the value and limitations of SNA and RC for measuring coordination of care as well as for learning from healthcare team members’ on-the-ground experience with epilepsy care coordination	Connectivity between neurologists and primary care providers as well as between neurologists and mental health providers were higher within ECOE hub facilities compared to spoke referring facilities
Sullivan 2019	To explore how knowledge, attitudes and behaviors diffuse between individuals through different network structures within bounded teams of trainee doctors	Clinical-technical behaviors were spread in a dense network with rich horizontal peer-to-peer connections. Patient-centred behaviors were spread in a sparse network. Additionally, highly influential individuals for clinical technical memes were identified
Zhu 2019	To explore (Q1) what main keywords are used to describe medical adverse events; (Q2) what main topics related to medical adverse events are discussed in each harm level? and, (Q3) what behavior or reason patterns exist in medical adverse events reports in terms of the relations between keywords and ideas for each harm level?	SNA and latent Dirichlet allocation analyses were integrated and revealed that communication, information transfer, and inattentiveness were the most common problems reported in the medical adverse events data
Holtrop 2018	To answer the following questions: Do care managers play a key role in chronic disease management in the practice, as noted by other practice members? Does the prominence of the care managers connectivity within the practice’s communication network vary by the type of care management structure implemented?	Care managers who were embedded in the practice or collocated were more likely to be in the core of the communication network than off-site care managers. These care managers also had higher in-degree centrality, indicating that they acted as a hub for communication with team members other roles
Bunger 2018	To examine change in professional advice-seeking patterns and factors that underlie the formation and maintenance of these ties among mental health clinicians participating in learning collaboratives to implement trauma-focused cognitive therapy (TF-CBT), an EBT for treating children with trauma-related mental health and behavioral problems	Participants formed/maintained advice-seeking relationships with those who possess perceived expertise and tended to seek advice from those within the same organization and with similar disciplinary training. Prior relationships and network structural features were associated with advice-seeking, indicating that participants built on existing social ties
Bunger 2018	To explore the changes in communication patterns within teams from children’s mental health organizations during a year-long learning collaborative focused on implementing a new treatment. We adopt a social network perspective to examine intra-organizational communication within each team and assess change in: (1) the frequency of communication among team members, (2) communication across organizational hierarchies, and (3) the overall structure of team communication networks	Individual level - participants reported communicating with more team members by the end of the learning collaborative. Team level - changes manifested depending on team size. Large teams: communication frequency increased, and networks grew denser and slightly less centralized. Small team: communication frequency declined, growing sparser and more centralized
Mundt 2015	To evaluate the associations between primary care team communication, interaction, and coordination (i.e., social networks); quality of care; and costs for patients with cardiovascular disease	Teams’ variations in communication patterns are associated with statistically significant differences in alcohol-related patient utilization and medical costs in their patient panels. Excessive alcohol-using patients fare better if they are cared for by teams with RNs who interact with LPNs/MAs and by teams whose frequent daily face-to-face communication to the primary care practitioner has been streamlined to a smaller number of team members
Wise 2015	To evaluate how differences in IT sophistication in nursing homes impact communication and use of technology related to skin care and pressure ulcers	High IT sophistication led to more diverse locations for healthcare worker interactions and low IT sophistication required more face-to-face interaction in more centralized locations within the nursing home
Stecher 2021	To estimate novel measures of generalist physicians’ network connectedness to HIV specialists and their associations with two dimensions of HIV quality of care	Generalists’ network connectedness to HIV specialists is positively related with their own HIV medication quality. A simulated policy that increases connectedness between generalists and HIV specialists reduces the annual rate of HIV infections by up to 6%. Only network connectedness to all physician types is associated with improved monitoring quality
Giorgio 2021	To address the shortcomings of the existing evidence on collaboration networks and organizational change in health organizations by exploring the following questions: In the context of structural changes, which characteristics of physicians past ties may foster or hinder the modification of their networks? How do physicians past ties affect their propensity to form cross-unit network relations?	Findings revealed that the physicians’ propensity to form cross-unit ties after the reorganization was related to the previous structure of their collaborative networks. The formation of cross-unit relations was negatively related to the size of advice networks before the reorganization. The diversity of network ties along with the presence of structural holes in the physicians’ networks before the change moderated this relationship
Stucky 2020	To characterize the typical OR communication patterns of clinicians at a military outpatient surgery center and determine how their interdependent relationships influence individual behavior	Communication effectiveness increased in networks which clinicians reported having close working relationships, socializing, and seeking advice and providing advice to others. Increases in individual clinician centrality were associated with increased communication effectiveness. Perioperative leaders should consider surgical team familiarity to optimize surgical care and improve communication effectiveness
Scott, 2005	To detail SNA measures that can be used to quantify patterns of decision-making and discuss how these measures could be used to facilitate the design and measure the outcomes of interventions to change organizational behavior in primary care practices	SNA measures allowed for the comparison of several aspects of decision-making patterns quantitatively.
Creswick, 2015	To identify and measure from whom hospital clinical staff seek medication advice on a weekly basis, quantify the use of other sources of medication information, assess differences in medication advice-seeking patterns across professional groups, and examine network characteristics in relation to prescribing error rates on 2 wards.	Physicians and nurses relatively rarely sought medication advice from each other. Nurses primarily sought advice from other nurses. Pharmacists, junior physicians, and senior nurses were identified as hubs of advice for ward staff. Junior physicians were crucial in providing medication advice to each other and to nurses.
Prusaczyk, 2019	To describe the communication networks of discharge planning teams for a cohort of older adults, and to examine the association of network characteristics with 30-day readmission.	Networks of patients not readmitted were more hierarchical, unidirectional, streamlined compared to those readmitted. These findings demonstrate the feasibility and usefulness of conceptualizing discharge planning as a network.
Alhaider, 2020	To apply the theory and methodology of DSA for (1) characterizing communication of system-wide patient flow managed by a command-and-control centre and (2) identifying communication deficiencies in the design and operation of the centre in facilitating patient flow.	Social network analysis provided centrality metrics to further characterize patient flow management. The Distributed Situation Awareness (DSA) model helped identify design principles and deficiencies in managing patient flow.
Lee, 2019	The objectives of this study were to describe the influence of peer-identified change agents (PICAs) and management-selected change agents (MSCAs) on hand hygiene, the perception of their leadership style by peers, their ability to shape team dynamics, and the role of the organizational culture in this process	Despite experiencing successful hand hygiene improvement from PICAs, HCWs expressed a preference for the existing top-down leadership structure. This highlights the limits of applying leadership models that are not supported by the local organizational culture.

**Table 3:** Summary of included study methodology

Author, date	Use of SNA	Data collection method	SNA metrics	Network Software
Hossain 2012	To create a regression model to understand the relationship and the influences that a social network has on coordination in the ED	Information system database / document artifact	Degree centrality; Density; Centralization	UCINET
Effken 2011	To examine the impact of unit level communication on patient safety and quality outcomes	Survey/questionnaire	Degree centrality; Betweenness centrality; Eigenvector centrality; Density; Cliques; Clustering coefficient; Simmelian ties; Fragmentation; Isolates	ORA
Benham-Hutchins 2010	To examine how network structure and communication can be used to design health information technology that compliments the nonlinear information gathering and dissemination behaviors of healthcare providers	Survey/questionnaire	Degree centrality; Betweenness centrality; Eigenvector centrality; Closeness centrality, Centralization	ORA
Salwei 2019	To understand team changes between low and high complexity stages	Interviews; Focus groups	Centralization; Density; Reciprocity	Lucidchart
Nengliang 2018	To describe the network structure for each patient and of the entire network and make comparisons between individual patients’ networks	Information system database / document artifact	Degree centrality; Betweenness centrality; Eigenvector centrality; Diameter; Clustering; Modularity	Gephi
Uddin 2012	To investigate physician collaboration network (PCN) using social network approach of network analysis to explore the impact of the attribute of PCN on hospitalization cost and readmission rate	Health insurance claim database	Degree centrality; Betweenness centrality; Density; Distance	UCINET
Parnell 2020	To explore associations of social structures (leadership) on hospital nursing units and nurse-sensitive outcomes (clinical indicators and measures of satisfaction) on those units	Survey/questionnaire	Degree centrality; Density; Centralization	UCINET
Grippa 2018	To map and measure the internal and external social networks to identify brokers, boundary spanners and central connectors who can transfer knowledge between departments and increase collaboration	Survey/questionnaire	Degree centrality; Betweenness centrality; Closeness centrality	None reported
Moore 2014	To explore how relational coordination and social network analysis could be used to inform the design and implementation of interventions intended to transform how disease management of diabetes is organized and delivered and how diabetes disease management providers work together to deliver care in support of the goals of health system reform	Survey/questionnaire	Degree centrality; Betweenness centrality; Eigenvector centrality	UCINET
Sarti 2020	To utilize social network mapping and analysis as an evaluative implementation measure to describe the physician network	Survey/questionnaire; Interviews	Degree centrality; Betweenness centrality	NVivo
Rangachari 2019	To describe the structure of inter-professional knowledge exchange on the SKN system	Online discussion board	Betweenness centrality	Not reported
Loveless 2015	To combine critical path analysis with mapping of the individual stroke team network interactions	Survey/questionnaire	None reported	Not reported
Bevc 2012	To understand interdependencies and communication flows in a public health system by examining the tendencies of network actors to fulfill specific roles within a network	Survey/questionnaire; Interviews	Degree centrality; Betweenness centrality	R software “sna” package
Samarth 2009	To find a relationship between group interaction patterns in the PACU and workflow processes	Survey/questionnaire	Degree centrality; Betweenness centrality; Density	CONDOR
Boyer 2010	To reveal which individuals are more “central” or “prestigious”, probably owing to their more influential positions, by virtue of their position in relation to other actors in this network locale	Survey/questionnaire	Prestige; Cliques; Centrality	UCINET
Rangachari 2008	To identify management and organizational characteristics associated hospital coding performance	Survey/questionnaire; Interviews	Density; betweenness centrality	UCINET
Westbrook 2007	To assess the impact of computerized order entry systems on both the technical and social systems within a health care organization	Survey/questionnaire; Observations	Degree centrality; Betweenness centrality	Not reported
Meltzer 2010	To demonstrate how social network principles can be applied to the design of quality improvement teams	Survey/questionnaire	Degree centrality; Betweenness centrality; Density	Pajek
Spitzer-Shohat 2019	To show that network structure and ties among primary care team members, working to improve the health and health care of their disadvantaged population groups, are related to the perceptions of their effectiveness	Survey/questionnaire; Interviews	Degree centrality; Betweenness centrality; Density	UCINET
Hurtado 2020	To identify influential workers who were then recruited, trained, and deployed as champions. We trained champions in QI to interact with formal leaders to act on issues germane to Safe patient handling and mobility	Survey/questionnaire	Centrality	UCINET
Steitz 2019	To quantify collaboration between providers by comparing physician messaging connectivity to patient sharing through outpatient appointments	Information system database / document artifact	Degree centrality; Density	R software “igraph” package
Pepin 2019	To demonstrate how social network analysis can visualize relationships which either facilitate or impede knowledge translation	Survey/questionnaire	Degree centrality; Density; Diameter	SocNetV
Altalib 2019	To explore individuals’ experiences with care coordination and develop new understanding of how and why RC and SNA are, or are not, similar and how each method can be used most effectively	Survey/questionnaire	Degree centrality; Betweenness centrality; Closeness centrality	Gephi
Sullivan 2019	To describe the social networks that diffuse knowledge, attitudes and behaviors relating to different domains of practice within teams of trainee doctors in an acute hospital medical setting	Survey/questionnaire; Interviews	Degree centrality; Betweenness centrality; Density	SocNetV
Zhu 2019	To compare primary medical adverse event keywords from reporters (e.g., physicians and nurses) and harm level perspectives to explore the underlying behaviors of medical adverse events using social network analysis (SNA) and latent Dirichlet allocation (LDA) leading to process improvements	Information system database / document artifact	Degree centrality; Betweenness centrality; Eigenvector centrality; Density	R software “igraph” package, Pajek
Holtrop 2018	To examine communication networks in primary care and illustrate the important role of care management structures in developing team-based care for chronic disease management	Survey/questionnaire	Degree centrality; Core periphery	VisuaLyzer
Bunger 2018	To understanding how clinicians seek and access expert advice over time	Survey/questionnaire	Density; Reciprocity; Transitivity; Centralization	R software “sna” package
Bunger 2018	To assess changes in the number of ties and frequency of communication among team members over the course of the learning collaborative	Survey/questionnaire	Degree centrality; Centralization	Not reported
Mundt 2015	To investigate which aspects of primary care team social networks are associated with higher quality of care and lower cost for patients with cardio-vascular disease	Survey/questionnaire; Interviews	Degree centrality; Density; Centralization	UCINET
Alexander 2015	To identify patterns in communication types and locations	Focus groups; Observations; previously collected survey data	None reported	ORA
Stecher 2021	To estimate the associations between connectedness to HIV specialists and these two dimensions of HIV quality of care (medication and monitoring quality) to test the ability and importance of specialist network connections for transferring complex medical knowledge to generalists	Information system database / document artifact	Degree centrality; betweenness centrality; Eigenvector centrality	R software “igraph” package
Giorgio 2021	To explore how the network structure affected the modification of network relations after reorganization	Survey/questionnaire	Betweenness centrality; Network size	UCINET
Stucky 2020	To present a characterization of individual clinician communication patterns and provide insight into how clinician interdependent relationships influence individual behavior	Survey/questionnaire	Degree centrality	UCINET
Scott, 2005	To describe how SNA can be used to characterize and compare communication patterns in primary care practices	Interviews; Observations	Centralization (in-degree / out-degree); Density; Hierarchy; Clustering coefficient	UCINET, KrackPlot
Creswick, 2015	To measure the weekly medication advice-seeking networks of hospital staff, to compare patterns across professional groups, and to examine these in the context of prescribing error rates.	Survey/questionnaire	Degree centrality; Density	UCINET
Prusaczyk, 2019	To utilize network analysis to identify the individuals involved in the discharge planning and their communication with each other for 205 patients.	Interviews; Information system database/document artifact	Density, Diameter	R software “igraph” package, Gephi
Alhaider, 2020	To model patient flow management of a healthcare provider to assess the merits of the theory and methodology for characterizing patient flow and identifying deficiencies	Interviews; Observations; Information system database / document artifact	Betweenness centrality; Closeness centrality	Gephi
Lee, 2019	This report is part of a study examining the effect of change agents on hand hygiene behaviour in acute healthcare	Survey/questionnaire	Degree centrality, Betweenness centrality, Density, Geodesic Distance, Reciprocity	NodeXLPro

**Table 4:** Summary of studies’ utilizing social network analysis (SNA) metrics

SNA Metrics	Definition	Number of Studies	SNA outcome
Degree Centrality	Total number of links in-coming and out-going in terms of volume of connections	26	• Measure of influence and importance – can identify a healthcare worker’s reach and ability to transfer information. • May influence performance e.g., too many connections for an individual may disrupt flow through interruptions or distractions, may be used to provide reinforcement for behaviors, and may be used to employ a shared idea (groupthink).
Density	Denotes the amount of linkage between actors (total connections / total possible connections)	20	• A high-density score indicates that there is a large amount of information sharing between all team members and could reflect complex procedures.
Betweenness Centrality	Indicates brokering and represents the shortest path connecting actors in a network	19	• Measure of influence and importance - identifies a worker’s potential to enhance or disrupt information flow, such as those in a gatekeeper role. • Highlights a provider who plays a central role in mediating or brokering communication among other individuals or groups.
Centralization	Extent to which the network relies on a single node or small number of nodes	8	• A low centralization score indicates there is a lot of interdependence between providers, whereas a high centralization indicates that one or a few roles are central to the team.
Eigenvector Centrality	Indicates individuals who are connected to highly connected people	6	• Measure of influence and importance – identifies a healthcare worker who is well-connected to other well-connected providers and can spread information quickly.
Closeness Centrality	The sum of the shortest paths between any two providers within a network	4	• Measure of influence and importance - identifies a healthcare worker who plays a central role in communicating with other providers either directly or along the shortest path.
Diameter	Refers to the longest of the possible paths between actors	3	• A large diameter implies that information (diffusion) does not spread easily in a network.
Clustering Coefficient	Extent to which there are small clusters (cliques)	3	• A higher clustering coefficient supports information diffusion and represents how information spreads across groups in the network.
Reciprocity	Measure of two-way communications and discussions in a network	3	• High reciprocity indicates there is a lot of communication and discussion among team members
Number of cliques	Sub-groups where the members are completely connected to each other	2	• Cliques can lead to limited dissemination of knowledge across the network through the creation of silos, which limit access to new opportunities.
Distance	Measures the minimum number of arcs between two actors	2	• Measure of healthcare worker embeddedness in a network
Core-periphery	Measure of whether an individual is in the denser and connected core versus the more sparse or less connected periphery	1	• High core–periphery may result when a core develops around certain healthcare providers who oversee many tasks, thus requiring other providers to interact with them.
Isolates	Number of nodes (individuals) that have no connections or links	1	• Measures the number of healthcare workers who have no connections or perform tasks alone.
Fragmentation	The proportion of entities (individuals) in a network that are disconnected	1	• Measures where silos exist in the network limiting communication across functional or hierarchical boundaries between providers.
Prestige	The extent to which an actor is contacted by others (the proportion of times each actor is “chosen” by others to discuss important subjects)	1	• Measure of influence and importance – identifies a healthcare worker who is important to work processes and may display leadership.
Simmelian ties	Number of strong ties embedded in cliques	1	• Providers who work closely together in small groups/teams can form strong reciprocative ties, which improve communication and information sharing.
Transitivity	Tendency toward triadic pathways (“a friend of a friend is a friend”)	1	• Providers are more likely to form and maintain ties with whom they have existing relationships or through a mutual third party.
Modularity	A measure of whether a network decomposes into subgroups.	1	• High modularity reflects an internal structure with significant crosslinking. Whereas low modularity indicates many separate clusters.
Hierarchy	The degree to which all relations are unidirectional.	1	• Measures the extent to which the transitive closure of the directed graph lacks symmetric ties.

^*^Some articles were assigned to more than one category.

Listed in descending frequency, however “Other” is always at the bottom.

## Application and findings of SNA

SNA has been used in healthcare to measure the number of connections (i.e., interactions, tasks), the centrality of providers (i.e., degree, betweenness, and closeness), and network cohesion (i.e., density, clustering). It has helped us to understand essential themes like organizational structure, team performance, and influential actors in healthcare.

a) Organizational Structure.

SNA has been used to better understand how organizational structures (e.g., management roles, groupings of tasks and employees) influence communication and coordination, thereby informing opportunities for improvement. Nine studies showed how SNA was used to redesign hospital organizational structures [35, 36, 41, 45, 46, 53, 66, 69, 72]. For example, Samarth et al. [69] applied SNA to improve the throughput of their surgical patients, which revealed a hierarchical network coordination structure in their post-anesthesia care unit (PACU) wherein the Charge Nurse channeled all communication downstream, thereby becoming a bottleneck resulting in patient delays. This led to a redesign of their organizational network to a more democratic structure where coordination was performed by an integrated information technology (IT) system which was available to all team members, reducing the dependence on the charge nurse [69]. Additionally, Alhaider et al. [52] demonstrated how SNA could be used to investigate system-wide communication in patient flow management and identify process improvement within the healthcare system. Applying SNA within the Distributed Situation Awareness (DSA) framework helped identify bottlenecks in patient flow and the roles that were most likely to experience communication or transaction overload while acquiring and disseminating situational awareness. The DSA model provided a characterization of patient flow and a blueprint for healthcare facilities to consider when modifying their organizational structure to improve communication and coordination. Spitzer-Shohat et al. [36] used SNA to understand how their organizational structure could help implement disparity reduction interventions to improve care. The SNA unveiled that their subregional management had a high degree of centrality (i.e., many connections), and as such, they were targeted to spread information about the interventions [36].

A specialized application of SNA involves identifying how IT can enhance or transform organizational communication and coordination. Three studies used SNA to understand how providers from different professions and units communicate across various modes (e.g., in-person, phone, electronic medical record) [4, 48, 69]. For example, SNA highlighted that IT could help improve communication efficiencies during in-person patient handoffs. More specifically, SNA showed that IT could support the redesign of the social network patterns by removing redundant communication exchanges and support emergent and non-linear information flow [4, 69]. Six studies used electronic health records (EHR) data to map the network structure of professionals involved in care to show that improving the design of IT can support communication leading to more frequent information sharing among professional groups [6, 47, 51, 56, 60, 63]. Nengliang et al. [56] demonstrated that EHR log data could be used within an SNA to map the network structure of all healthcare providers and examine the connectivity, centrality, and clustering of networks that emerged from interactions between providers who shared patients. In turn, this data revealed the dynamic nature of care teams and areas (inpatient and outpatient) for collaborative improvement [56]. Another study used SNA to help contrast low and high IT implementations; they found that the high IT sophistication care homes had more robust and integrated communication strategies requiring fewer face-to-face interactions between providers to verify orders or report patient status compared to the low IT sophistication nursing home [47].

b) Team Performance.

Sixteen studies used SNA to examine poor team communication and coordination by highlighting the inefficiencies in health networks [3, 36, 41, 43, 53–55, 57, 58, 61, 64, 65, 67, 68, 70, 71]. SNA identified that these inefficiencies stem from: teams being overburdened due to workload [54, 61], conflict between team roles [36], lack of leadership [43, 58], and fragmented interprofessional relationships [57, 65, 70]. For example, poor team performance in hospital emergency departments has resulted in congestion and increased length of stay with patients having prolonged discharges. SNA allowed for an exploration of the possible causes of inefficiencies resulting in access blocks and determined that the number of healthcare providers and interactions between them, and the centralization of providers within the network affected the performance and quality of emergency departments [54]. Grippa et al. [3] used SNA and determined that the most efficient and effective healthcare teams focused more inwardly (internal team operation) and were less connected to external members. Additionally, SNA highlighted that effective teams communicated using only one or two mediums (e.g., in-person, email, instant messaging media) instead of dispersing time on multiple media applications.

SNA has been used to diagnose possible reasons for team inefficiencies and to identify potential design solutions to improve team performance [3, 35, 42, 53, 64, 67, 68, 71]. A study used SNA to identify that some experienced staff (who frequently mentor other staff) may have too many connections (high degree of centrality), leading to interruptions or distractions and impacting performance and coordination [54]. However, a different study, identified that staff with a high degree of centrality have the benefit of improving team performance by leveraging their social networks to be change agents and lead others to replicate desired behaviors (e.g., when a provider may forget to implement a desired change but gets reminded by a team member) [62]. Lastly, analyzing network cohesion helped identify fragmentation and cliques in the network which may reflect a lack of collaboration and interprofessional relations. For instance, denser (more connections) communication networks with more clustering (groups of connections) are associated with more rapid diffusion of information. Additionally, the connections between providers in dense networks can provide social support (reinforcement) to team members that strengthen their commitment to follow desired behaviors and increase the likelihood that deviations from those actions will be noted by their peers [62].

c) Influential Actors.

SNA was used to identify influential actors who could act as brokers (an individual who occupies a specific structural position in systems of exchange) [3, 49, 64] who could become opinion leaders (an individual who holds significant influence over others’ attitudes/beliefs) [62], champions (an individual who actively supports innovation and its promotion/implementation) [40] or a change agent (an early adopter of an intervention who supports the dissemination of its use) [44] based off measures of social influence within a network. Studies showed that influential actors in social networks can inform behavioral interventions needed to improve professional communication or coordination [3, 40, 49, 62, 64]. For example, Meltzer et al. [62] used SNA to identify influential physicians to join a QI team and highlighted that having members with connections external to the team is most important when disseminating information, while within team relationships matter most when coordination, knowledge sharing, and within-group communication are most important. When creating an interdisciplinary team, betweenness centrality (node that frequently lies on the shortest path in a network) may be a useful network metric for prospectively identifying team members that may help to facilitate coordination within and across units / professional groups. Providers with a high betweenness have been found to be leaders and active participants in task-related groups [68]. Hurtado et al. [40] used SNA to identify and recruit champions who were used to deploy a QI intervention (safe patient handling education program) to advance safety in critical access hospitals. The champion-centered approach resulted in improved safety outcomes (increase in safety participation/compliance and decrease in patient-assist injuries) after one year. Additionally, Lee et al. [44] used SNA to assess the use of peer-identified and management-selected change agents on improving hand hygiene behavior in acute healthcare. No significant differences were reported between the two groups; however providers expressed a preference for hierarchical leadership styles highlighting the need to understand organizational culture before designing changes to the system.

## Discussion

This scoping review presents a comprehensive overview of the existing literature looking at the use and impact of SNA methodology on Process Improvement within healthcare organizations. Our search strategy included a wide range of databases and placed no restrictions on study design, language, or publication period. When examining the expanding body of literature represented in our identified 38 studies, SNA methods were used to detect essential work processes in organizations, reveal bottlenecks in workflow, offer insight into resource allocation, evaluate team performance, identify influential providers, and monitor the effectiveness of process improvements over time. By analyzing the communication and relationships between management roles, employee groupings, and task allocation, SNA provides insights that can help identify areas for improvement related to patient throughput, diffusion of information, and the uptake of technology (e.g., IT systems). Studies highlighted that healthcare team performance can be hampered by inefficiencies related to being overburdened due to workload, conflicts between team roles, lack of leadership, and fragmented interprofessional relationships. To address these inefficiencies, SNA can leverage network outcomes related to connectedness (e.g., degree, betweenness, closeness) and use knowledge of the network structure (e.g., density, clustering coefficient, fragmentation) to create targeted interventions to mitigate these problems. Additionally, inefficiencies in social networks can be mitigated by identifying influential actors who serve as change agents and can be utilized as opinion leaders or champions to improve the efficiency of information exchange and the uptake of behavioral interventions.

Comparison With Past Literature (Study Design and Data Collection).

Our review stands out from previous studies due to its unique focus on the application of SNA methods in Process Improvement within healthcare organizations. Our primary objective was to investigate how healthcare organizations utilize SNA techniques to improve system-level coordination and enhance the overall quality of care provided to patients. In their research study, Sabot et al. [22] aimed to investigate the various SNA methods employed to examine professional communication and performance among healthcare professionals. Their study delved into the diverse range of SNA techniques used to gain insights into the complex network dynamics and interactions among providers. In more recent studies, Saatchi et al. [78] focused on exploring the adoption and implementation of network interventions in healthcare settings. This study provided insights into the effectiveness of network interventions (in which contexts they are successful and for whom), their potential benefits (increased volume of communication), and the challenges associated with their adoption in practice. Additionally, Rostami et al. [79] focused on advancing quantitative SNA techniques and investigated the application of community detection algorithms in healthcare. This study offers a comprehensive categorization of SNA community detection algorithms and explores potential approaches to overcome gaps and challenges in their use. Previous reviews primarily included observational and cross-sectional study designs with no comparator arms, which made determining the value of using SNA methods difficult as there was no comparison of social networks over time and no comparable head-to-head data. Our review identified 5 quasi-experimental studies [40–44] which used longitudinal or pre-post study designs. In each of these studies SNA was used to review a system which delivered clinical care to identify sources of variation and areas for process improvement at an individual and organizational level. The quasi-experimental studies were published within the last 5 years, indicating that SNA methodology is still in development and opportunities for experimental and longitudinal study designs are forthcoming. Using experimental and longitudinal SNA methods would enable causal inference of healthcare interventions or policies leading to improved generalizability of results.

When performing SNA there is a variety of qualitative (interviews, focus groups, observations) and quantitative (surveys, document artifacts, information systems) methods that researchers can use to map social networks, assess network structures, and analyze team actors. However, previous literature reviews have outlined an overreliance on descriptive SNA methods, which lack the contextual factors needed to interpret how a network reached a given structure. There has been a growing body of evidence advocating for the use of mixed-method social network data collection [80]. Our review has highlighted an increased uptake of mixed-method (integration of qualitative and quantitative methods and data) and multi-method (independent use of quantitative and qualitative methods) SNA study designs [81].

Knowledge Gaps and Future Research.

This scoping review highlights many practical uses of SNA; however, within most studies, little attention has been paid to leveraging SNA theory to help explain why networks have the structures they do [21]. For example, social boundaries between professional groups (e.g., Physicians, Nurses, Pharmacists) can inhibit the development of interprofessional networks though the creation of cliques leading to strong communication and coordination within groups, but fragmented communication across professional groups [21, 82, 83]. A potential explanation for the scarcity of studies assessing the reasons behind the structures of networks could be attributed to the primarily quantitative SNA methods used. Few studies used a qualitative or mixed-method design, indicating a limited understanding of the contextual factors associated with social networks. SNA can reveal the informal structures within organizations and underscores the importance of understanding that not all influential relationships between healthcare providers are found on formal organizational charts, and that informal networks can significantly influence communication and coordination [84]. The lack of robust study designs (mixed-method or multi-method) may also reflect the use of SNA by researchers more so as a technique than a methodology with theoretical underpinnings.

The value of using SNA to inform research and disseminate evidence-based interventions and policies has been discussed in the literature extensively. However, very few studies have used research on complex systems and network theory to examine how HCWs can act as change agents, interacting within and between hubs in organizations to disseminate knowledge [85]. Future research should apply complexity science to SNA to reconceptualize knowledge translation and think of the process as interdependent and relationship-centric to support sustainable translation [85]. Only a small group of included articles have highlighted how leveraging influential actors as change agents such as opinion leaders or champions can be advantageous in improving professional communication or coordination [3, 40, 44, 49, 62, 64]. This review identified two studies [40, 44] which utilized SNA and a champion-centered approach to support the successful implementation of a QI intervention resulting in improved safety outcomes. The use of champions is very prevalent in healthcare; however, success rates vary widely, likely due to the poor selection of champion candidates or organizational culture [40, 44]. In many cases healthcare workers selected to be champions are volunteered and do not hold enough social influence to change the behaviors of their colleagues. In the future SNA methods should be used to identify influential champions or opinion leaders embedded within their social networks who can influence knowledge transfer and facilitate coordination leading to process improvements.

Future research should identify how SNA methods can leverage health informatics and the large amounts of data stored within healthcare organizations. Even though past studies have used SNA to enhance organizational communication and coordination using IT [47, 56, 69], applying SNA to artificial intelligence and machine learning (ML) algorithms has not received much attention [86]. Integrating ML algorithms into community detection techniques has showcased the diverse ways SNA can be utilized in healthcare to monitor disease diagnosis, track outbreaks, and analyze HCW networks [79].

Limitations of the Review.

This review has some limitations that should be acknowledged. First, we excluded studies of provider friendship networks, which theoretically may have contained some professional communication. Secondly, we excluded studies where network relations were defined solely by patient sharing, as this has only been shown to predict person-to-person communication in a minority of instances. Lastly, studies were required to incorporate a Process Improvement component. Different terms were used to describe Process Improvement in the literature, making it challenging to devise a search strategy that would yield sufficient articles for review while also utilizing SNA methods. As a result, studies that utilized SNA methods but did not explicitly examine a process or system for delivering clinical care to identify sources of variation and areas for improvement were excluded.

## Conclusion

SNA methods can be used to characterize Process Improvements through mapping, quantifying, and visualizing social relations revealing inefficiencies, which can then be targeted to develop interventions to enhance communication, foster collaboration, and improve patient safety. However, healthcare organizations still lack an understanding of the benefit of using SNA methods to reduce adverse events due to a lack of experimental studies. By emphasizing the importance of understanding professional communication and coordination within healthcare teams, units, and organizations, our review underscores the relationship between organizational structures and the potential of influential actors and emerging IT technologies to mitigate adverse events and improve patient safety.

## Electronic supplementary material

Below is the link to the electronic supplementary material.

Supplementary Material 1

Supplementary Material 2

Supplementary Material 3

## Data Availability

All data generated or analyzed during this study are included in this published article [and its supplementary information files].
