# Supplementary material for: Exploring the use of social network analysis methods in process improvement within healthcare organizations: a scoping review
Source: BMC Health Serv Res. 2024 Sep 5;24:1030. doi: 10.1186/s12913-024-11475-1 (PMC11376022; doi:10.1186/s12913-024-11475-1)
Supplement: Supplementary file 1 — Supplementary Material 1 [file 12913_2024_11475_MOESM1_ESM.docx]

**Exploring the Use of Social Network Analysis Methods in Process Improvement Within Healthcare Organizations: A Scoping Review**

**Study Inclusion Checklist**

| **Participants (Healthcare Providers):** |  |  |
| --- | --- | --- |
| - Are the study participants healthcare providers? OR   - physicians, nurses, midwives, pharmacists, clinical officers, counselors, and others who provide health-related services to patients in medical environments - Others involved in their professional social networks?   - administrative, support and secretarial staff | **YES** | **NO** |
| **Setting (Healthcare facilities):** |  |  |
| - Hospitals, nursing homes, limited care facilities, clinics, medical and dental offices, or ambulatory care centers | **YES** | **NO** |
| **Intervention (Social Network Analysis & Quality Improvement):** |  |  |
| - Does the study use Social Network Analysis methods?   - Social network mapping,   - Assessment of network structure and properties, or   - Analysis of network members | **YES** | **NO** |
| - Does the study provide recognized features of Quality Improvement?   - use of data analysis to assess and review the effect of changes,   - review of a process or system used to deliver clinical care to identify sources of variation and areas for improvement,   - Involvement of iterative development and testing,   - A structured process improvement method that is used to plan and test changes to work processes | **YES** | **NO** |
| **Outcomes reported:** |  |  |
| - Is the communication/relationship of interest between healthcare providers? | **YES** | **NO** |
| - Does the research focus on professional communication or inter-dependent tasks? | **YES** | **NO** |
| - Does the study report Social Network Analysis results (e.g. centrality, cohesion scores or SNA mapping) | **YES** | **NO** |
| **Study inclusion:** |  |  |
| - All the answers are YES | **INCLUDE** | |
| - Any answer is NO | **EXCLUDE** | |

| **Category** | **Inclusion Criteria** | **Exclusion Criteria** |
| --- | --- | --- |
| Participants | Are the study participants healthcare providers or administrative, support and secretarial staff? | Studies examining institution-to-institution communication and relationships |
| Setting | Does the study take place in a healthcare organization? |  |
| Intervention | Does the study use SNA methods? | Social network methods were mentioned, but analyses were not performed |
|  | Does the study provide recognized features of Continuous Quality Improvement? (i.e., Lean Management, Six Sigma, Plan-Do-Study-Act (PDSA) cycles, and Root Cause Analysis) |  |
| Outcome | Is the communication/relationship of interest between healthcare providers? | Studies evaluating patient-patient or patient-provider communication |
|  | Does the research focus on professional communication or inter-dependent tasks? |  |
|  | Does the study report Social Network Analysis results (e.g. centrality, degree, cohesion scores) | No SNA analyses and results are reported |
