# Supplementary material for: Exploring the use of social network analysis methods in process improvement within healthcare organizations: a scoping review
Source: BMC Health Serv Res. 2024 Sep 5;24:1030. doi: 10.1186/s12913-024-11475-1 (PMC11376022; doi:10.1186/s12913-024-11475-1)
Supplement: Supplementary file 3 — Supplementary Material 3 [file 12913_2024_11475_MOESM3_ESM.docx]

**Title: Exploring the Use of Social Network Analysis Methods in Process Improvement Within Healthcare Organizations: A Scoping Review**

**Date of updated searches:** 16 October 2022

**Database searched:**

- Ovid Medline
- Ovid Embase
- Ovid PsycINFO
- Ovid AMED
- Cochrane CENTRAL
- CINAHL
- Web of Science

**Limits:**

Humans

**MULTIFILE SEARCH**

Database(s): **Ovid MEDLINE: Epub Ahead of Print, In-Process & Other Non-Indexed Citations, Ovid MEDLINE® Daily and Ovid MEDLINE®**1946-Present**, Embase Classic+Embase**1947-Present

Search Strategy:

| **#** | **Searches** | **Results** |
| --- | --- | --- |
| 1 | Social Network Analysis/ or (((social$ or peer) adj4 network$ adj4 (analy$ or approach or data$ or diagram$ or examin$ or explor$ or framework$ or indicator$ or information or inquir$ or interaction$ or investigat$ or intervention$ or map$ or measure$ or method$ or metric? or model? or pattern? or perspective$ or questionnaire$ or software? or structure$ or survey$ or technique$ or theor$ or tool$)) or ((egocentric or sociocentric or ego-centric or socio-centric or whole) adj2 network?)).tw,kw,kf. | 19769 |
| 2 | (exp Interprofessional Relations/ or Community-Institutional Relations/ or Hospital-Physician Relations/ or Interdepartmental Relations/ or Interinstitutional Relations/ or Interprofessional Relations/ or Community Networks/ or Group Structure/ or (((relations$ or dynamics) adj3 (interprofessional or institutional or hospital-physician or interdepartmental or interinstitutional or community-institutional)) or (community adj2 (care or health or healthcare))).tw,kw,kf.) and (network or networks).tw,kw,kf. | 15532 |
| 3 | exp Sociometric techniques/ or (sociometr$ or sociogram$ or sociomap$ or network structure).tw,kw,kf. | 19147 |
| 4 | 2 and 3 | 186 |
| 5 | (UCINET or NetDraw or KrackPlot or NetMiner or StOCNET or GRADAP or NEGOPY or FATCAT or MultiNet or Agna or Blue Spider or DyNet or MDLogix Solutions or Network Workbench or Pajek or Sentinel Visualizer or SocNetV or visone or CID-ABM or C-IKNOW or Commetrix or MetaSight or Referral Web or SONIVIS or CiteSpace or E-Net or Ego Net or VennMaker or Financial Network Analyzer or PGRAPH or PermNet or CFinder or KeyPlayer or KliqFinder or Network Genie or ONA surveys or NodeXL or MatMan or yFiles or LibSNA or NetworkX or UrlNet or igraph or latentnet or RSiena or statnet or tnet).tw,kw. | 1729 |
| 6 | 1 or 4 or 5 [SOCIAL NETWORK ANALYSIS TERMS] | 21274 |
| 7 | Total Quality Management/ or Workflows/ or Work Performance/ or Quality Control/ or Process Assessment, Health Care/ or Program Evaluation/ or Program Development/ or Benchmarking/ or Quality Assurance, Health Care/ or Total Quality Management/ or Quality Improvement/ or Quality Indicators, Health Care/ or Health Care Evaluation Mechanisms/ or ((quality adj2 (assurance or improvement? or collaborative? or manag$ or control? or indicator? or metric? or assessment? or evaluation?)) or workflow? or work-flow? or (performance? adj2 (work or job or vocational)) or (quality adj2 ((continuous$ or total) adj2 (manag$ or improv$))) or CQI or TQM or ((process or processes or system?) adj2 (improv$ or redesign$ or assessment? or measure?)) or (program$ adj2 (develop$ or plan$ or description? or evaluat$ or sustainabilit$ or effectiveness or appropriateness)) or benchmarking or bench-marking or (social adj2 validity) or ((quality improvement? or QI or QA) adj3 (team? or microsystem? or micro-system? or cycle?)) or PDSA or PCDA or TQIS or ((shewhart or shewart or deming) adj3 (cycle? or method?)) or (breakthrough adj3 (series or project? or collaborative?)) or (lean adj (approach?? or management or method? or thinking or enterpri#e or practice or philosoph$ or principle?)) or (organi?ational adj2 change?) or (model? adj2 improvement) or (((rapid adj cycle?) or "iterative cycle" or Kaizen or Kansei) and quality) or "Global Trigger Tool" or "six sigma" or Taguchi method? or "plan do study" or "plan do check" or "Plan-Do-Study-Act" or "Plan-Do-Check-Act" or "business process reengineering" or "define-measure-analyse-improve-control" or "define-measure-analyse-design-verify" or "quality function deployment" or "House of quality" or "Toyota production system" or "lean manufacturing" or "continuous quality improvement" or "statistical process control" or "team quality improvement sequence").tw,kw. or ("root cause" or "value stream" or "practice change").tw. [QUALITY IMPROVEMENT TERMS] | 1367170 |
| 8 | 6 and 7 | 934 |
| 9 | exp Animals/ not (exp Animals/ and Humans/) [ANIMAL-ONLY REMOVED] | 16929661 |
| 10 | 8 not 9 | 750 |
| 11 | 10 use ppez | 410 |
| 12 | (((social$ or peer) adj4 network$ adj4 (analy$ or approach or data$ or diagram$ or examin$ or explor$ or framework$ or indicator$ or information or inquir$ or interaction$ or investigat$ or intervention$ or map$ or measure$ or method$ or metric? or model? or pattern? or perspective$ or questionnaire$ or software? or structure$ or survey$ or technique$ or theor$ or tool$)) or ((egocentric or sociocentric or ego-centric or socio-centric or whole) adj2 network?)).tw,kw,hw. | 19551 |
| 13 | (social network/ or public relations/ or community care/ or Group Structure/ or (((relations$ or dynamics) adj3 (interprofessional or institutional or hospital-physician or interdepartmental or interinstitutional or community-institutional)) or (community adj2 (care or health or healthcare))).tw,kw,kf.) and (network or networks).tw,kw,hw. | 32883 |
| 14 | sociometric status/ or (sociometr$ or sociogram$ or sociomap$ or network structure).tw,kw,hw. | 19064 |
| 15 | 13 and 14 | 740 |
| 16 | (UCINET or NetDraw or KrackPlot or NetMiner or StOCNET or GRADAP or NEGOPY or FATCAT or MultiNet or Agna or Blue Spider or DyNet or MDLogix Solutions or Network Workbench or Pajek or Sentinel Visualizer or SocNetV or visone or CID-ABM or C-IKNOW or Commetrix or MetaSight or Referral Web or SONIVIS or CiteSpace or E-Net or Ego Net or VennMaker or Financial Network Analyzer or PGRAPH or PermNet or CFinder or KeyPlayer or KliqFinder or Network Genie or ONA surveys or NodeXL or MatMan or yFiles or LibSNA or NetworkX or UrlNet or igraph or latentnet or RSiena or statnet or tnet).tw,kw. | 1729 |
| 17 | 12 or 15 or 16 [SOCIAL NETWORK ANALYSIS TERMS] | 21167 |
| 18 | total quality management/ or workflow/ or job performance/ or quality control/ or health care quality/ or social validity/ or performance measurement system/ or program evaluation/ or program development/ or total quality management/ or quality of care research/ or ((quality adj2 (assurance or improvement? or collaborative? or manag$ or control? or indicator? or metric? or assessment? or evaluation?)) or workflow? or work-flow? or (performance? adj2 (work or job or vocational)) or (quality adj2 ((continuous$ or total) adj2 (manag$ or improv$))) or CQI or TQM or ((process or processes or system?) adj2 (improv$ or redesign$ or assessment? or measure?)) or (program$ adj2 (develop$ or plan$ or description? or evaluat$ or sustainabilit$ or effectiveness or appropriateness)) or benchmarking or bench-marking or (social adj2 validity) or ((quality improvement? or QI or QA) adj3 (team? or microsystem? or micro-system? or cycle?)) or PDSA or PCDA or TQIS or ((shewhart or shewart or deming) adj3 (cycle? or method?)) or (breakthrough adj3 (series or project? or collaborative?)) or (lean adj (approach?? or management or method? or thinking or enterpri#e or practice or philosoph$ or principle?)) or (organi?ational adj2 change?) or (model? adj2 improvement) or (((rapid adj cycle?) or "iterative cycle" or Kaizen or Kansei) and quality) or "Global Trigger Tool" or "six sigma" or Taguchi method? or "plan do study" or "plan do check" or "Plan-Do-Study-Act" or "Plan-Do-Check-Act" or "business process reengineering" or "define-measure-analyse-improve-control" or "define-measure-analyse-design-verify" or "quality function deployment" or "House of quality" or "Toyota production system" or "lean manufacturing" or "continuous quality improvement" or "statistical process control" or "team quality improvement sequence").tw,kw. or ("root cause" or "value stream" or "practice change").tw. [QUALITY IMPROVEMENT TERMS] | 1369225 |
| 19 | 17 and 18 | 916 |
| 20 | (exp animal/ or exp animal experimentation/ or exp animal model/ or exp animal experiment/ or nonhuman/ or exp vertebrate/) not (exp human/ or exp human experimentation/ or exp human experiment/) [ANIMAL-ONLY REMOVED] | 12366782 |
| 21 | 19 not 20 | 896 |
| 22 | 21 use emczd [Embase results] | 510 |
| 23 | 11 or 22 [MEDLINE and Embase results combined] | 920 |
| **24** | **remove duplicates from 23 [MEDLINE and Embase results deduplicated]** | **629** |

**PsychINFO Search**

Search Strategy:

| **#** | **Searches** | **Results** |
| --- | --- | --- |
| 1 | Social Network Analysis/ or (((social$ or peer) adj4 network$ adj4 (analy$ or approach or data$ or diagram$ or examin$ or explor$ or framework$ or indicator$ or information or inquir$ or interaction$ or investigat$ or intervention$ or map$ or measure$ or method$ or metric? or model? or pattern? or perspective$ or questionnaire$ or software? or structure$ or survey$ or technique$ or theor$ or tool$)) or ((egocentric or sociocentric or ego-centric or socio-centric or whole) adj2 network?)).mp. | 12559 |
| 2 | (exp Social Networks/ or Professional Networking/ or Public Relations/ or Intergroup Dynamics/ or Interpersonal Interaction/ or Collaboration/ or Cooperation/ or Teamwork/ or Employee Interaction/ or exp Organizational Behavior/ or exp Group Dynamics/ or Interdependence/ or Peer Relations/ or Communities of Practice/ or (((relations$ or dynamics) adj3 (interprofessional or institutional or hospital-physician or interdepartmental or interinstitutional or community-institutional)) or social or peer? or collaborat$ or cooperat$ or organization$ or (community adj2 (care or health or healthcare))).mp.) and (network or networks).mp. | 75004 |
| 3 | exp Sociometry/ or (sociometr$ or sociogram$ or sociomap$ or network structure).mp. | 7772 |
| 4 | 2 and 3 | 1794 |
| 5 | (UCINET or NetDraw or KrackPlot or NetMiner or StOCNET or GRADAP or NEGOPY or FATCAT or MultiNet or Agna or Blue Spider or DyNet or MDLogix Solutions or Network Workbench or Pajek or Sentinel Visualizer or SocNetV or visone or CID-ABM or C-IKNOW or Commetrix or MetaSight or Referral Web or SONIVIS or CiteSpace or E-Net or Ego Net or VennMaker or Financial Network Analyzer or PGRAPH or PermNet or CFinder or KeyPlayer or KliqFinder or Network Genie or ONA surveys or NodeXL or MatMan or yFiles or LibSNA or NetworkX or UrlNet or igraph or latentnet or RSiena or statnet or tnet).mp. | 228 |
| 6 | 1 or 4 or 5 [SOCIAL NETWORK ANALYSIS TERMS] | 13570 |
| 7 | Quality Control/ or Organizational Effectiveness/ or "Quality of Care"/ or "Quality of Services"/ or Group Performance/ or Productivity/ or Organizational Effectiveness/ or Job Performance/ or Program Evaluation/ or Program Development/ or ((quality adj2 (assurance or improvement? or collaborative? or manag$ or control? or indicator? or metric? or assessment? or evaluation?)) or workflow? or work-flow? or (performance? adj2 (work or job or vocational)) or (quality adj2 ((continuous$ or total) adj2 (manag$ or improv$))) or CQI or TQM or ((process or processes or system?) adj2 (improv$ or redesign$ or assessment? or measure?)) or (program$ adj2 (develop$ or plan$ or description? or evaluat$ or sustainabilit$ or effectiveness or appropriateness)) or benchmarking or bench-marking or (social adj2 validity) or ((quality improvement? or QI or QA) adj3 (team? or microsystem? or micro-system? or cycle?)) or PDSA or PCDA or TQIS or ((shewhart or shewart or deming) adj3 (cycle? or method?)) or (breakthrough adj3 (series or project? or collaborative?)) or (lean adj (approach?? or management or method? or thinking or enterpri#e or practice or philosoph$ or principle?)) or (organi?ational adj2 change?) or (model? adj2 improvement) or (((rapid adj cycle?) or "iterative cycle" or Kaizen or Kansei) and quality) or "Global Trigger Tool" or "six sigma" or Taguchi method? or "plan do study" or "plan do check" or "Plan-Do-Study-Act" or "Plan-Do-Check-Act" or "business process reengineering" or "define-measure-analyse-improve-control" or "define-measure-analyse-design-verify" or "quality function deployment" or "House of quality" or "Toyota production system" or "lean manufacturing" or "continuous quality improvement" or "statistical process control" or "team quality improvement sequence").mp. or ("root cause" or "value stream" or "practice change").tw. [QUALITY IMPROVEMENT TERMS] | 178236 |
| 8 | 6 and 7 | 618 |
| 9 | exp Animals/ | 359507 |
| **10** | **8 not 9 [ANIMAL-ONLY REMOVED]** | **616** |

**Allied Complementary Medicine Database (AMED) Search**

Search Strategy:

| **#** | **Searches** | **Results** |
| --- | --- | --- |
| 1 | (((social$ or peer) adj4 network$ adj4 (analy$ or approach or data$ or diagram$ or examin$ or explor$ or framework$ or indicator$ or information or inquir$ or interaction$ or investigat$ or intervention$ or map$ or measure$ or method$ or metric? or model? or pattern? or perspective$ or questionnaire$ or software? or structure$ or survey$ or technique$ or theor$ or tool$)) or ((egocentric or sociocentric or ego-centric or socio-centric or whole) adj2 network?)).mp. | 126 |
| 2 | (exp Interprofessional Relations/ or Interinstitutional Relations/ or Interprofessional Relations/ or Public Relations/ or Professional Patient Relations/ or Peer Group/ or (((relations$ or dynamics) adj3 (interprofessional or institutional or hospital-physician or interdepartmental or interinstitutional or community-institutional)) or social or peer? or collaborat$ or cooperat$ or organization$ or (community adj2 (care or health or healthcare))).mp.) and (network or networks).mp. | 851 |
| 3 | (sociometr$ or sociogram$ or sociomap$ or network structure).mp. | 31 |
| 4 | 2 and 3 | 5 |
| 5 | (UCINET or NetDraw or KrackPlot or NetMiner or StOCNET or GRADAP or NEGOPY or FATCAT or MultiNet or Agna or Blue Spider or DyNet or MDLogix Solutions or Network Workbench or Pajek or Sentinel Visualizer or SocNetV or visone or CID-ABM or C-IKNOW or Commetrix or MetaSight or Referral Web or SONIVIS or CiteSpace or E-Net or Ego Net or VennMaker or Financial Network Analyzer or PGRAPH or PermNet or CFinder or KeyPlayer or KliqFinder or Network Genie or ONA surveys or NodeXL or MatMan or yFiles or LibSNA or NetworkX or UrlNet or igraph or latentnet or RSiena or statnet or tnet).mp. | 4 |
| 6 | 1 or 4 or 5 [SOCIAL NETWORK ANALYSIS TERMS] | 131 |
| 7 | ((Quality Control/ or Quality Assurance Health Care/ or Outcome.mp.) and Process Assessment/) or Program Evaluation/ or Program Development/ or "Quality of health care"/ or ((quality adj2 (assurance or improvement? or collaborative? or manag$ or control? or indicator? or metric? or assessment? or evaluation?)) or workflow? or work-flow? or (performance? adj2 (work or job or vocational)) or (quality adj2 ((continuous$ or total) adj2 (manag$ or improv$))) or CQI or TQM or ((process or processes or system?) adj2 (improv$ or redesign$ or assessment? or measure?)) or (program$ adj2 (develop$ or plan$ or description? or evaluat$ or sustainabilit$ or effectiveness or appropriateness)) or benchmarking or bench-marking or (social adj2 validity) or ((quality improvement? or QI or QA) adj3 (team? or microsystem? or micro-system? or cycle?)) or PDSA or PCDA or TQIS or ((shewhart or shewart or deming) adj3 (cycle? or method?)) or (breakthrough adj3 (series or project? or collaborative?)) or (lean adj (approach?? or management or method? or thinking or enterpri#e or practice or philosoph$ or principle?)) or (organi?ational adj2 change?) or (model? adj2 improvement) or (((rapid adj cycle?) or "iterative cycle" or Kaizen or Kansei) and quality) or "Global Trigger Tool" or "six sigma" or Taguchi method? or "plan do study" or "plan do check" or "Plan-Do-Study-Act" or "Plan-Do-Check-Act" or "business process reengineering" or "define-measure-analyse-improve-control" or "define-measure-analyse-design-verify" or "quality function deployment" or "House of quality" or "Toyota production system" or "lean manufacturing" or "continuous quality improvement" or "statistical process control" or "team quality improvement sequence").mp. or ("root cause" or "value stream" or "practice change").tw. [mp=abstract, heading words, title] | 13867 |
| 8 | 6 and 7 | 6 |
| 9 | exp Animals/ not (exp Animals/ and Humans/) | 13697 |
| **10** | **8 not 9 [ANIMAL-ONLY REMOVED]** | **6** |

**CINAHL Plus**

Interface - EBSCOhost Research Databases
Search Screen - Advanced Search
Database - CINAHL Plus with Full Text

| **#** | **Query** | **Results** |
| --- | --- | --- |
| S10 | S8 NOT S9 | 3,518 |
| S9 | (MH "Vertebrates+") not ((MH "Vertebrates+") and (MH "Human")) | 203,553 |
| S8 | S6 AND S7 | 3,523 |
| S7 | (MH "Quality Management, Organizational") OR (MH "Quality Improvement") OR (MH "Benchmarking") OR (MH "Root Cause Analysis") OR (MH "Variance Analysis") OR (MH "Workflow") OR (MH "Job Performance") OR (MH "Quality Management, Organizational") OR (MH "Process Assessment (Health Care)") OR (MH "Quality Assessment") OR (MH "Process Assessment (Health Care)") OR (MH "Program Evaluation") OR (MH "Program Development") OR TX ((quality N2 (assurance or improvement? or collaborative? or manag* or control? or indicator? or metric? or assessment? or evaluation?)) or workflow? or work-flow? or (performance? N2 (work or job or vocational)) or (quality N2 ((continuous* or total) N2 (manag* or improv*))) or CQI or TQM or ((process or processes or system?) N2 (improv* or redesign* or assessment? or measure?)) or (program* N2 (develop* or plan* or description? or evaluat* or sustainabilit* or effectiveness or appropriateness)) or benchmarking or bench-marking or (social N2 validity) or ((quality improvement? or QI or QA) N3 (team? or microsystem? or micro-system? or cycle?)) or PDSA or PCDA or TQIS or ((shewhart or shewart or deming) N3 (cycle? or method?)) or (breakthrough N3 (series or project? or collaborative?)) or (lean N (approach?? or management or method? or thinking or enterpri#e or practice or philosoph* or principle?)) or (organi?ational N2 change?) or (model? N2 improvement) or (((rapid N cycle?) or "iterative cycle" or Kaizen or Kansei) and quality) or "Global Trigger Tool" or "six sigma" or Taguchi method? or "plan do study" or "plan do check" or "Plan-Do-Study-Act" or "Plan-Do-Check-Act" or "business process reengineering" or "define-measure-analyse-improve-control" or "define-measure-analyse-design-verify" or "quality function deployment" or "House of quality" or "Toyota production system" or "lean manufacturing" or "continuous quality improvement" or "statistical process control" or "team quality improvement sequence") or TI ("root cause" or "value stream" or "practice change") or AB ("root cause" or "value stream" or "practice change") | 484,088 |
| S6 | S1 OR S4 OR S5 | 12,185 |
| S5 | TX (UCINET or NetDraw or KrackPlot or NetMiner or StOCNET or GRADAP or NEGOPY or FATCAT or MultiNet or Agna or Blue Spider or DyNet or MDLogix Solutions or Network Workbench or Pajek or Sentinel Visualizer or SocNetV or visone or CID-ABM or C-IKNOW or Commetrix or MetaSight or Referral Web or SONIVIS or CiteSpace or E-Net or Ego Net or VennMaker or Financial Network Analyzer or PGRAPH or PermNet or CFinder or KeyPlayer or KliqFinder or Network Genie or ONA surveys or NodeXL or MatMan or yFiles or LibSNA or NetworkX or UrlNet or igraph or latentnet or RSiena or statnet or tnet) | 667 |
| S4 | S2 AND S3 | 1,003 |
| S3 | (MH "Sociometric Techniques+") or TX (sociometr* or sociogram* or sociomap* or network structure) | 21,227 |
| S2 | ((MH "Interprofessional Relations+") or (MH "Community-Institutional Relations") or (MH "Hospital-Physician Joint Ventures") or (MH "Interdepartmental Relations") or (MH "Interinstitutional Relations") or (MH "Interprofessional Relations") or (MH "Community Networks") or (MH "Public Relations") or TX (((relations* or dynamics) N3 (interprofessional or institutional or hospital-physician or interdepartmental or interinstitutional or community-institutional)) or (community N2 (care or health or healthcare)))) AND TX (network or networks) | 47,339 |
| S1 | (MH "Social Network Analysis") or TX (((social* or peer) N4 network* N4 (analy* or approach or data* or diagram* or examin* or explor* or framework* or indicator* or information or inquir* or interaction* or investigat* or intervention* or map* or measure* or method* or metric? or model? or pattern? or perspective* or questionnaire* or software? or structure* or survey* or technique* or theor* or tool*)) or ((egocentric or sociocentric or ego-centric or socio-centric or whole) N2 network?))) | 10,95 |

**Cochrane Library**

ID Search Hits

#1 MeSH descriptor: [Social Network Analysis] this term only 2

#2 ((((social* or peer) NEAR/4 network* NEAR/4 (analy* or approach or data* or diagram* or examin* or explor* or framework* or indicator* or information or inquir* or interaction* or investigat* or intervention* or map* or measure* or method* or metric? or model? or pattern? or perspective* or questionnaire* or software? or structure* or survey* or technique* or theor* or tool*)) or ((egocentric or sociocentric or ego-centric or socio-centric or whole) NEAR/2 network?))):ti,ab,kw 784

#3 #1 OR #2 784

#4 MeSH descriptor: [Interprofessional Relations] explode all trees 577

#5 MeSH descriptor: [Community-Institutional Relations] this term only 188

#6 MeSH descriptor: [Hospital-Physician Relations] this term only 3

#7 MeSH descriptor: [Interdepartmental Relations] this term only 0

#8 MeSH descriptor: [Interinstitutional Relations] this term only 46

#9 MeSH descriptor: [Interprofessional Relations] this term only 300

#10 MeSH descriptor: [Community Networks] this term only 170

#11 MeSH descriptor: [Group Structure] this term only 30

#12 ((((relations* or dynamics) NEAR/3 (interprofessional or institutional or hospital-physician or interdepartmental or interinstitutional or community-institutional)) or (community NEAR/2 (care or health or healthcare)))):ti,ab,kw 9773

#13 {OR #4-#12} 10170

#14 ((network or networks)):ti,ab,kw 15827

#15 #13 AND #14 603

#16 MeSH descriptor: [Sociometric Techniques] explode all trees 31

#17 ((sociometr* or sociogram* or sociomap* or network structure)):ti,ab,kw 538

#18 #16 OR #17 539

#19 #15 AND #18 20

#20 ((UCINET or NetDraw or KrackPlot or NetMiner or StOCNET or GRADAP or NEGOPY or FATCAT or MultiNet or Agna or Blue Spider or DyNet or MDLogix Solutions or Network Workbench or Pajek or Sentinel Visualizer or SocNetV or visone or CID-ABM or C-IKNOW or Commetrix or MetaSight or Referral Web or SONIVIS or CiteSpace or E-Net or Ego Net or VennMaker or Financial Network Analyzer or PGRAPH or PermNet or CFinder or KeyPlayer or KliqFinder or Network Genie or ONA surveys or NodeXL or MatMan or yFiles or LibSNA or NetworkX or UrlNet or igraph or latentnet or RSiena or statnet or tnet)):ti,ab,kw 391

#21 #1 OR #19 OR #20 412

#22 MeSH descriptor: [Total Quality Management] this term only 139

#23 MeSH descriptor: [Workflow] this term only 76

#24 MeSH descriptor: [Work Performance] this term only 49

#25 MeSH descriptor: [Quality Control] this term only 476

#26 MeSH descriptor: [Process Assessment, Health Care] this term only 253

#27 MeSH descriptor: [Program Evaluation] this term only 6270

#28 MeSH descriptor: [Program Development] this term only 725

#29 MeSH descriptor: [Benchmarking] this term only 110

#30 MeSH descriptor: [Quality Assurance, Health Care] this term only 608

#31 MeSH descriptor: [Quality Improvement] this term only 752

#32 MeSH descriptor: [Quality Indicators, Health Care] this term only 227

#33 MeSH descriptor: [Health Care Evaluation Mechanisms] this term only 0

#34 (((quality NEAR/2 (assurance or improvement? or collaborative? or manag* or control? or indicator? or metric? or assessment? or evaluation?)) or workflow? or work-flow? or (performance? NEAR/2 (work or job or vocational)) or (quality NEAR/2 ((continuous* or total) NEAR/2 (manag* or improv*))) or CQI or TQM or ((process or processes or system?) NEAR/2 (improv* or redesign* or assessment? or measure?)) or (program* NEAR/2 (develop* or plan* or description? or evaluat* or sustainabilit* or effectiveness or appropriateness)) or benchmarking or bench-marking or (social NEAR/2 validity) or ((quality improvement? or QI or QA) NEAR/3 (team? or microsystem? or micro-system? or cycle?)) or PDSA or PCDA or TQIS or ((shewhart or shewart or deming) NEAR/3 (cycle? or method?)) or (breakthrough NEAR/3 (series or project? or collaborative?)) or (lean NEAR (approach?? or management or method? or thinking or enterpri#e or practice or philosoph* or principle?)) or (organi?ational NEAR/2 change?) or (model? NEAR/2 improvement) or (((rapid NEAR cycle?) or "iterative cycle" or Kaizen or Kansei) and quality) or "Global Trigger Tool" or "six sigma" or Taguchi method? or "plan do study" or "plan do check" or "Plan-Do-Study-Act" or "Plan-Do-Check-Act" or "business process reengineering" or "define-measure-analyse-improve-control" or "define-measure-analyse-design-verify" or "quality function deployment" or "House of quality" or "Toyota production system" or "lean manufacturing" or "continuous quality improvement" or "statistical process control" or "team quality improvement sequence")):ti,ab,kw 41661

#35 (("root cause" or "value stream" or "practice change")):ti 26

#36 (("root cause" or "value stream" or "practice change")):ab 291

#37 {OR #22-#36} 41888

#38 #21 AND #37 82

**Web of Science:** **BKCI-S, ESCI, CPCI-S, SCI-EXPANDED**

Science Citation Index Expanded (SCI-EXPANDED)

Conference Proceedings Citation Index – Science (CPCI-S)

Social Sciences Citation Index (SSCI)

Emerging Sources Citation Index (ESCI)

Conference Proceedings Citation Index – Social Science & Humanities (CPCI-SSH)

Book Citation Index – Science (BKCI-S)

Book Citation Index – Social Sciences & Humanities (BKCI-SSH) (Web of Science Index)

<https://www.webofscience.com/wos/woscc/summary/f2558680-ab67-40d3-963b-a1cf994cdd8e-01a15287/relevance/1>

| **#** | **Searches** |  |
| --- | --- | --- |
| 1 | TS=((((social* or peer) NEAR/4 network* NEAR/4 (analy* or approach or data* or diagram* or examin* or explor* or framework* or indicator* or information or inquir* or interaction* or investigat* or intervention* or map* or measure* or method* or metric? or model? or pattern? or perspective* or questionnaire* or software? or structure* or survey* or technique* or theor* or tool*)) or ((egocentric or sociocentric or ego-centric or socio-centric or whole) NEAR/2 network?))) | 36,362 |
| 2 | (TS=((((relations* or dynamics) NEAR/3 (interprofessional or institutional or hospital-physician or interdepartmental or interinstitutional or community-institutional)) or (community NEAR/2 (care or health or healthcare))) and (network or networks))) AND TS=((sociometr* or sociogram* or sociomap* or network structure)) | 561 |
| 3 | TS=((UCINET or NetDraw or KrackPlot or NetMiner or StOCNET or GRADAP or NEGOPY or FATCAT or MultiNet or Agna or Blue Spider or DyNet or MDLogix Solutions or Network Workbench or Pajek or Sentinel Visualizer or SocNetV or visone or CID-ABM or C-IKNOW or Commetrix or MetaSight or Referral Web or SONIVIS or CiteSpace or E-Net or Ego Net or VennMaker or Financial Network Analyzer or PGRAPH or PermNet or CFinder or KeyPlayer or KliqFinder or Network Genie or ONA surveys or NodeXL or MatMan or yFiles or LibSNA or NetworkX or UrlNet or igraph or latentnet or RSiena or statnet or tnet)) | 4,605 |
| 4 | ((#1) OR #2) OR #3[SOCIAL NETWORK ANALYSIS TERMS] | 41,026 |
| 5 | TS=(("Global Trigger Tool" or "six sigma" or "Taguchi method?" or "plan do study" or "plan do check" or "Plan-Do-Study-Act" or "Plan-Do-Check-Act" or "business process reengineering" or "define-measure-analyse-improve-control" or "define-measure-analyse-design-verify" or "quality function deployment" or "House of quality" or "Toyota production system" or "lean manufacturing" or "continuous quality improvement" or "statistical process control" or "team quality improvement sequence") or ("root cause" or "value stream" or "practice change")) | 33,695 |
| 6 | TS=((quality NEAR/2 (assurance or improvement? or collaborative? or manag* or control? or indicator? or metric? or assessment? or evaluation?)) or workflow? or work-flow? or (performance? NEAR/2 (work or job or vocational)) or (quality NEAR/2 ((continuous* or total) NEAR/2 (manag* or improv*))) or CQI or TQM or ((process or processes or system?) NEAR/2 (improv* or redesign* or assessment? or measure?)) or (program* NEAR/2 (develop* or plan* or description? or evaluat* or sustainabilit* or effectiveness or appropriateness)) or benchmarking or bench-marking or (social NEAR/2 validity) or (("quality improvement?" or QI or QA) NEAR/3 (team? or microsystem? or micro-system? or cycle?)) or PDSA or PCDA or TQIS or ((shewhart or shewart or deming) NEAR/3 (cycle? or method?)) or (breakthrough NEAR/3 (series or project? or collaborative?)) or (lean NEAR/1 (approach?? or management or method? or thinking or enterpri$e or practice or philosoph* or principle?)) or (organi?ational NEAR/2 change?) or (model? NEAR/2 improvement) or (((rapid NEAR/1 cycle?) or "iterative cycle" or Kaizen or Kansei) and quality)) | 667,218 |
| 7 | (#5) OR #6 | 691,968 |
| 8 | (#4) AND #7 | [1,383](https://www-webofscience-com.myaccess.library.utoronto.ca/wos/woscc/summary/f2558680-ab67-40d3-963b-a1cf994cdd8e-01a15287/relevance/1) |
| 9 | (#4) AND #7 and Computer Science Information Systems or Computer Science Theory Methods or Computer Science Artificial Intelligence or Engineering Electrical Electronic or Telecommunications or Computer Science Interdisciplinary Applications or Computer Science Hardware Architecture or Computer Science Software Engineering or Environmental Sciences or Information Science Library Science or Education Educational Research or Engineering Industrial or Engineering Civil or Mathematics Interdisciplinary Applications or Engineering Multidisciplinary or Mathematical Computational Biology or Physics Multidisciplinary or Education Scientific Disciplines or Environmental Studies or Computer Science Cybernetics or Green Sustainable Science Technology or Business or Construction Building Technology or Mathematics Applied or Ecology or Engineering Environmental or Imaging Science Photographic Technology or Physics Mathematical or Robotics or Water Resources or Engineering Manufacturing or Hospitality Leisure Sport Tourism or Materials Science Multidisciplinary or Transportation Science Technology or Agriculture Multidisciplinary or Communication or Energy Fuels or Agronomy or Engineering Mechanical or Geography or Humanities Multidisciplinary or Veterinary Sciences or Business Finance or Geosciences Multidisciplinary or Mechanics or Meteorology Atmospheric Sciences or Optics or Physics Applied or Physics Fluids Plasmas or Remote Sensing or Agricultural Economics Policy or Biology or Instruments Instrumentation or Physics Condensed Matter or Biodiversity Conservation or Chemistry Analytical or Forestry or History Philosophy Of Science or Marine Freshwater Biology or Mathematics or Political Science or Regional Urban Planning or Transportation or Zoology or Acoustics or Agricultural Engineering or Agriculture Dairy Animal Science or Astronomy Astrophysics or Chemistry Multidisciplinary or Chemistry Physical or Electrochemistry or Engineering Aerospace or Engineering Chemical or Geochemistry Geophysics or Horticulture or Linguistics or Logic or Metallurgy Metallurgical Engineering or Oceanography or Philosophy or Soil Science or Thermodynamics or Urban Studies **(Exclude – Web of Science Categories)** | 371 |
